# Reciprocal Association between the Apical Junctional Complex and AMPK: A Promising Therapeutic Target for Epithelial/Endothelial Barrier Function?

**DOI:** 10.3390/ijms20236012

**Published:** 2019-11-29

**Authors:** Kazuto Tsukita, Tomoki Yano, Atsushi Tamura, Sachiko Tsukita

**Affiliations:** 1 Laboratory of Biological Science, Graduate School of Frontier Biosciences, Osaka University, Suita, Osaka 565-0811, Japan; kazusan@kuhp.kyoto-u.ac.jp (K.T.); atamura@biosci.med.osaka-u.ac.jp (A.T.); 2 Department of Neurology, Graduate School of Medicine, Kyoto University, Kyoto 606-8507, Japan; 3 Laboratory of Biological Science, Graduate School of Medicine, Osaka University, Suita, Osaka 565-0811, Japan; t-yano@biosci.med.osaka-u.ac.jp; 4 Strategic Innovation and Research Center, Teikyo University, Tokyo 173-8605, Japan

**Keywords:** tight junction, adherens junction, apical junctional complex, AMP-activated protein kinase (AMPK), paracellular barrier

## Abstract

Epithelial/endothelial cells adhere to each other via cell–cell junctions including tight junctions (TJs) and adherens junctions (AJs). TJs and AJs are spatiotemporally and functionally integrated, and are thus often collectively defined as apical junctional complexes (AJCs), regulating a number of spatiotemporal events including paracellular barrier, selective permeability, apicobasal cell polarity, mechano-sensing, intracellular signaling cascades, and epithelial morphogenesis. Over the past 15 years, it has been acknowledged that adenosine monophosphate (AMP)-activated protein kinase (AMPK), a well-known central regulator of energy metabolism, has a reciprocal association with AJCs. Here, we review the current knowledge of this association and show the following evidences: (1) as an upstream regulator, AJs activate the liver kinase B1 (LKB1)–AMPK axis particularly in response to applied junctional tension, and (2) TJ function and apicobasal cell polarization are downstream targets of AMPK and are promoted by AMPK activation. Although molecular mechanisms underlying these phenomena have not yet been completely elucidated, identifications of novel AMPK effectors in AJCs and AMPK-driven epithelial transcription factors have enhanced our knowledge. More intensive studies along this line would eventually lead to the development of AMPK-based therapies, enabling us to manipulate epithelial/endothelial barrier function.

## 1. Introduction

Epithelial/endothelial cells adhere to each other via cell–cell junctions, thereby collectively functioning as a permselective barrier between the internal and external environments of the body to define the framework of every biological compartment of our body [1,2,3,4,5,6]. Because the size and function of each compartment and their inter-relationships are critical for biological systems, the specific and general principles regulating the permselective barrier function of epithelial/endothelial cells are of considerable interest. Additionally, it is attractive from a therapeutic point of view to investigate methods to manipulate barrier function, given that its disruption can lead to various diseases [7,8,9,10,11,12,13,14,15,16,17]. For example, changes in paracellular barrier function can initiate and accelerate inflammation (both locally and systemically) [10,11,12,15,16,17], contribute to tumor formation [14,18], initiate infection [19], and disrupt ion homeostasis [9,13,20], and have even been suggested as being potentially involved in the initiation and progression of psychiatric and neurodegenerative diseases [21,22,23,24,25,26].

Tight junctions (TJs) are vertebrate-specific junctions that are located most apically in epithelial cell–cell junctions. It is generally accepted that adherens junctions (AJs), which are located immediately at the basal side of TJs, are essential for epithelial cell sheet formation, on the basis of which TJs create a paracellular permselective barrier [6,27,28]. Therefore, TJs and AJs are considered to be spatiotemporally and functionally integrated, and are thus often collectively known as apical junctional complexes (AJCs). In addition to creating a paracellular barrier, AJCs play essential roles in apicobasal cell polarity, mechano-sensing, intracellular signaling cascades, and epithelial morphogenesis [1,6,28,29,30,31,32]. A number of signaling networks are involved in AJC-related events and, over the past 15 years, adenosine monophosphate (AMP)-activated protein kinase (AMPK), a well-known central regulator of energy metabolism signaling pathway, has been shown to have a reciprocal association with AJCs [33,34,35,36,37,38,39,40]. More specifically, AJs activate AMPK particularly in response to applied junctional tension, whereas TJ barrier function and apicobasal cell polarization are promoted by AMPK activation. Herein, we review the critical studies regarding this reciprocal association and discuss the therapeutic potential of AMPK to manipulate epithelial/endothelial barrier function.

## 2. Overview of Function and Regulation of AMPK

In the 1970s and 1980s, a series of biochemical experiments identified the presence of a 3-hydroxy-3-methylglutaryl-coenzyme A (HMG-CoA) reductase kinase, which was found to be activated by AMP [41,42,43]. Meanwhile, an acetyl-coenzyme A (acetyl-CoA) carboxylase kinase was purified from rat liver in the mid-1980s and was also found to be activated by AMP [44]. Later, the independently identified HMG-CoA reductase kinase and acetyl-CoA carboxylase kinase were shown to be the same protein, and this protein was subsequently given the name “AMPK” after its characteristic feature of activation by AMP [45,46].

**Figure 1:**
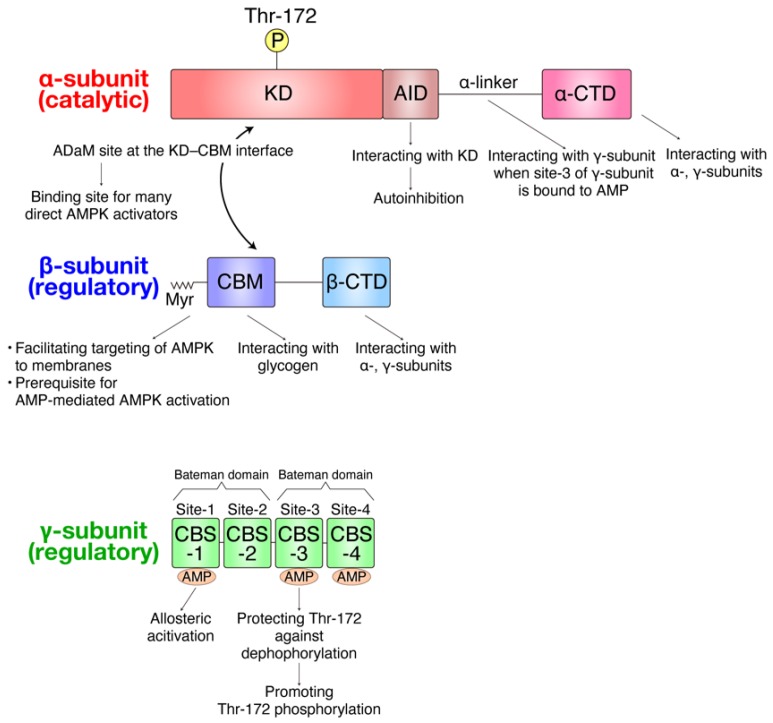
Domains of adenosine monophosphate (AMP)-activated protein kinase (AMPK) subunits. The kinase domain (KD) in the catalytic α-subunit exerts AMPK kinase activity, and Thr-172 phosphorylation of the KD is critical for its activity. The autoinhibitory domain (AID) binds to the backside of KD, constraining its mobility, thereby inhibiting kinase activity [52]. When AMP is bound at site-3 in the γ-subunit, the α-linker binds to the γ-subunit, thereby pulling AID away from the KD [53]. The C-terminal domain of the α-subunit (α-CTD) is responsible for interacting with the β- and γ-subunits. The allosteric drug and metabolite (ADaM) site at the KD–carbohydrate-binding module (CBM) interface is a binding site for many direct AMPK activators that induces allosteric activation, including A-769662 and compound 991 (see Table 1) [56]. The N-terminal of the regulatory β-subunit has been shown to be myristoylated, which facilitates targeting of AMPK to membranes and is prerequisite for AMP-mediated α-subunit Thr-172 phosphorylation [54]. The CBM in the β-subunit interacts with glycogen, which facilitates targeting of AMPK to glycogen for glycogen turnover regulation [55]. The C-terminal domain of the β-subunit (β-CTD) interacts with both α- and γ- subunits and serves as a connection between the α- and γ- subunits [51]. The regulatory γ-subunits contain four cystathionine beta synthase (CBS) domains, packing together to generate two Bateman domains, thereby providing four potential AMP-binding sites (site-1 to site-4) [48]. However, in mammalian AMPK, only site-1, site-3, and site-4 bind to AMP because aspartic acid residues in a conserved potential binding site in site-2 are changed to arginine [48]. Site-4 contains a permanently-bound AMP molecule, whereas AMP transiently binds to site-1 and site-3 (site-1 has a much stronger binding affinity for AMP than site-3). AMP binding to site-1 causes allosteric activation of AMPK, and AMP binding to site-3 promotes α-subunit Thr-172 phosphorylation by protecting AMPK from upstream phosphatases [49]. Myr, myristoylation.

AMPK is a heterotrimeric serine/threonine kinase composed of one catalytic α-subunit and two regulatory β- and γ-subunits, and is highly conserved in all eukaryotes from protozoa to vertebrates (Figure 1) [47,48,49,50,51,52,53,54,55,56].

**Figure 2:**
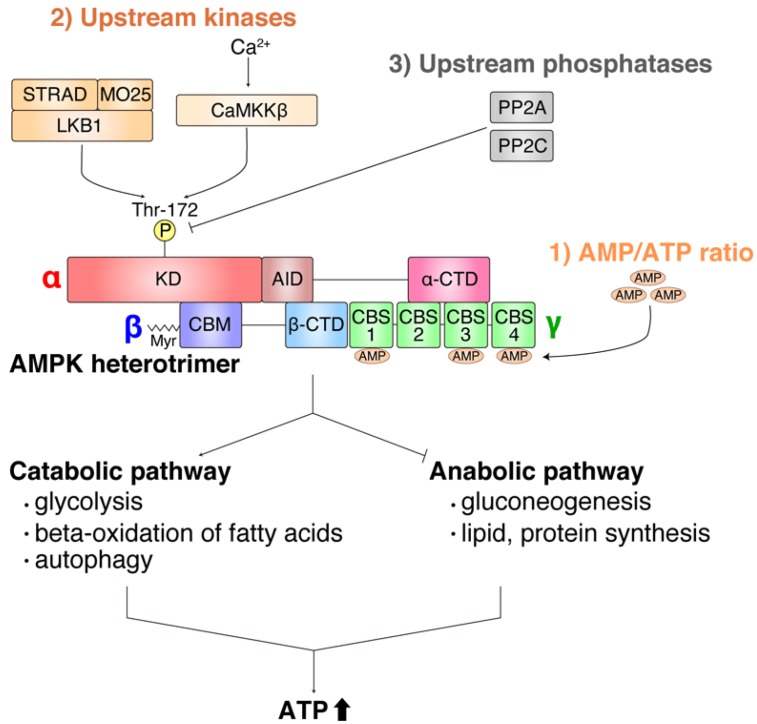
Overview of adenosine monophosphate (AMP)-activated protein kinase (AMPK) activation and the biological consequences. Activation of AMPK is determined by the balance of (1) AMP/adenosine triphosphate (ATP) ratio, (2) upstream kinase activity, and (3) upstream phosphatase activity. Once activated, AMPK activates catabolic pathways such as glycolysis, beta-oxidation of fatty acids, and autophagy, as well as suppresses anabolic pathway such as gluconeogenesis and lipid/protein synthesis to increase ATP levels. LKB1, liver kinase B1; STRAD, ste-20 related adaptor; MO25, mouse protein 25; CaMKKβ, CaMKK Ca^2+^/calmodulin-dependent protein kinase kinase β; PP2A, protein phosphatase 2A; PP2C, protein phosphatase 2C; KD, kinase domain; AID, auto-inhibitory domain; CTD, C-terminal domain; CBM, carbohydrate-binding module; Myr, myristoylation; CBS, cystathionine beta synthase.

AMPK functions as a central regulator of energy metabolism and, once activated, it turns on catabolic processes as well as turns off anabolic processes, leading to upregulation of adenosine triphosphate (ATP) concentrations (Figure 2). Activation of AMPK is determined by the balance of the following three factors (Figure 2) [48,49,57]: (1) the AMP/ATP ratio [48,57], (2) the activity of upstream kinases to phosphorylate α-subunit Thr-172 including liver kinase B1 (LKB1) and Ca^2+^/calmodulin-dependent protein kinase kinase β (CaMKKβ) [58,59], and (3) the activity of upstream protein phosphatases that dephosphorylate α-subunit Thr-172 such as protein phosphatase 2A and 2C [49,60].

**Figure 3:**
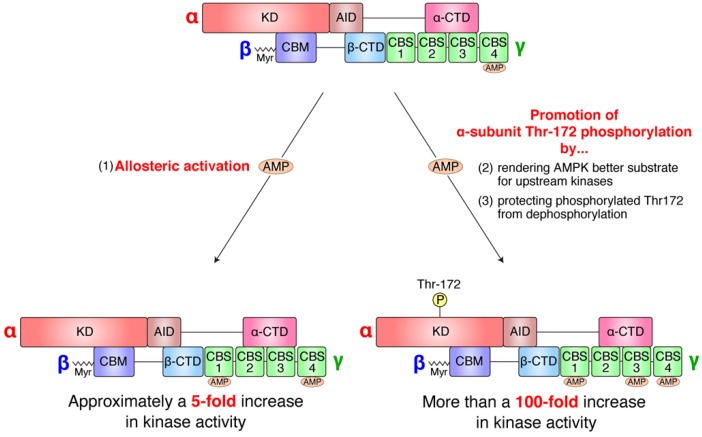
Adenosine monophosphate (AMP) activates AMP-activated protein kinase (AMPK) in several different ways. Allosteric activation results in approximately a fivefold increase in kinase activity, whereas α-subunit Thr-172 phosphorylation causes more than a 100-fold increase in kinase activity; therefore, α-subunit Thr-172 phosphorylation is essentially considered to be a molecular on–off switch for AMPK. KD, kinase domain; AID, auto-inhibitory domain; CTD, C-terminal domain; CBM, carbohydrate-binding module; Myr, myristoylation; CBS, cystathionine beta synthase.

Notably, binding of AMP to the γ-subunit can activate AMPK by three mechanisms that can be antagonized by ATP (Figure 3): (1) AMP drives direct allosteric activation of AMPK [61], (2) AMP renders AMPK susceptible to α-subunit Thr-172 phosphorylation by upstream kinases [62,63], and (3) AMP protects phosphorylated α-subunit Thr-172 from dephosphorylation by upstream phosphatases [64]. It should also be noted that the effect of direct allosteric activation by AMP on AMPK activity is modest (approximately a fivefold increase in activity) [61] compared with that of α-subunit Thr-172 phosphorylation (more than 100-fold increase in activity) [64]; therefore, the phosphorylation status of α-subunit Thr-172 is essentially a molecular on–off switch for AMPK activity [57] (Figure 3). Because AMPK activation has potential to mediate a variety of different cellular consequences, AMPK activation is controlled, not only temporally by the phosphorylation status of α-subunit Thr-172, but also spatially [47,57]. Spatially, AMPK is mobilized to various subcellular organelles only in response to appropriate signals. As examples, glucose starvation leads to AMPK targeting to lysosomes, increased autophagic influx results in AMPK targeting to autophagosomes, and mitochondrial damage causes AMPK targeting to mitochondria [65].

## 3. Notable Findings in the Mid-2000s Established the Important Role of the LKB1–AMPK Axis in Apicobasal Epithelial Cell Polarity

In 2004, Clevers and colleagues shocked the epithelial cell research community with their seminal paper [33]. Until then, it was believed that apicobasal epithelial cell polarity was established after cells formed cell–cell junctions [66]. However, using intestinal epithelial cell lines with inducible LKB1 activity, they showed that, even in the absence of junctional cell–cell contacts, activation of LKB1 alone can fully establish apicobasal cell polarity in a cell-autonomous manner with the three major aspects of cell polarity all fulfilled, these being the appropriate sorting of apical and basolateral plasma membrane markers, formation of a full brush border, and correct junctional localization of junctional proteins. LKB1 forms a complex with ste-20 related adaptor (STRAD) and mouse protein 25 (MO25) that exerts many functions through the phosphorylation of many substrates [67]. In 2005, the downstream LKB1 effector responsible for establishing apicobasal cell polarity was suggested to be AMPK by investigating the functional consequences of a missense mutation identified in Peutz–Jeghers syndrome (PJS) that is caused by germline mutations of the LKB1 gene [34]. It was found that there is a certain LKB1-C-terminal missense mutation found in PJS patients that abolish the kinase activity responsible for almost all effects of the LKB1/STRAD/MO25 complex but still impair AMPK activity and apicobasal polarity establishment [34]. Subsequently, in 2007, it was found that not only *Drosophila* LKB1-null mutants but also *Drosophila* AMPK-null mutants display severe abnormalities in apicobasal cell polarity [36]. These findings collectively showed the crucial role of the LKB1–AMPK axis in apicobasal epithelial cell polarity establishment and have certainly paved the way for subsequent elucidation of the intimate association between AMPK and AJCs.

## 4. AJs and Junctional Tension Promote the LKB1–AMPK Axis to Provide Energy to Resist Applied Force

**Figure 4:**
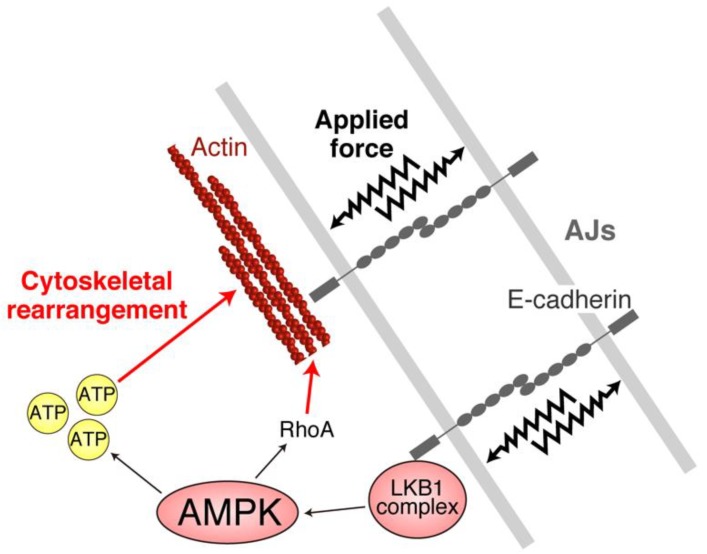
Adherens junctions (AJs) activate the liver kinase B1 (LKB1)–AMP-activated protein kinase (AMPK) axis. AJs can promote LKB1 complex activation [38], which is facilitated by applied force (zigzag arrows) [39]. Activated LKB1 complexes subsequently activate AMPK, which increases ATP levels and promotes RhoA activation to rearrange cytoskeletons, providing stiffness to resist against applied force.

Interestingly, in 2009, it was found that AJs can activate LKB1 complexes [38]. In this study, it was shown that active LKB1 complexes co-localize with E-cadherin in polarized Caco-2 and Madin–Darby canine kidney (MDCK) II cells, that E-cadherin-dependent junctional maturation gradually mobilizes LKB1 complexes to AJs, and that AJ localization of LKB1 complexes is critical for the activation of AMPK. However, the physiological relevance of the LKB1–AMPK axis regulation at AJs remained unknown until 2017 when this regulation was shown to be critical for epithelial cells to resist against applied force. In epithelial cells, mechano-sensing, especially at AJs, triggers robust actin cytoskeletal rearrangements to resist externally applied forces [68,69,70]. From an energetic point of view, these changes are very costly, consuming about half of the total ATP in a cell. In 2017, it was found that junctional tension directly applied on E-cadherin, as well as shear stress on MDCK II cells, facilitates AJ-mediated LKB1–AMPK activation. Activated AMPK subsequently increases ATP levels and activates RhoA to support peri-junctional actomyosin filament rearrangement, thereby contributing to the cell stiffness critical for resisting against applied force [39] (Figure 4). In vivo importance of this AJ-mediated LKB1–AMPK regulation is suggested by the study of the tumor suppressor gene folliculin (FLCN), the causative gene for a rare autosomal-dominant disorder named Birt–Hogg–Dubé (BHD) syndrome. It was shown that FLCN deletion in lung epithelium results in downregulation of E-cadherin and LKB1 expression, which leads to the impairment of AMPK activation and alveolar collapse [71]. Collectively, these findings established the critical role of AJs and junctional tension in promoting the LKB1–AMPK axis to resist applied force.

## 5. TJ Function Is Reinforced by AMPK

The first report that showed the association of AMPK with TJs was published in 2006 [35]. At that time, AMPK was shown to facilitate apicobasal polarity establishment [34,36] and TJs have long been believed to function as a “fence” to prevent the diffusion of proteins and lipids, thereby having a role in apicobasal cell polarity [72] (later, “fence” function of TJs has been basically denied [73,74], although some controversial discussions appeared again recently [75]). Therefore, the role of AMPK in TJ assembly was investigated using a calcium switch experiment, a prevalent experiment to study TJ assembly. In a calcium switch experiment, calcium depletion from the medium of confluent epithelial cells destabilizes AJs, which triggers TJ disassembly with zonula occludens-1 (ZO-1) translocation from cell–cell junctions to the cytoplasm. Calcium restoration causes TJ assembly with ZO-1 redistribution from the cytoplasm to cell–cell junctions to form nascent ZO-1 puncta, which later forms continuous ZO-1 structures. Using this experimental procedure, it was shown that AMPK is activated during TJ assembly in MDCK II cells and that 5-aminoimidazole-4-carboxamide ribonucleoside (AICAR), a potent AMPK activating reagent, facilitates ZO-1 redistribution from the cytoplasm to cell–cell junctions, whereas expression of a dominant-negative AMPK (a kinase-dead form of AMPK) significantly delays ZO-1 redistribution with impaired establishment of the paracellular barrier. It was also noted that AMPK activation alone does not lead to the formation of continuous ZO-1 structures from puncta, suggesting that AMPK is required in the initiation of TJ assembly but other factors are needed to support further TJ maturation [35]. Shortly after this, it was also shown that AMPK activation by AICAR partially protects TJs from disassembly induced by calcium depletion [37]. Altogether, these studies established that TJs are a downstream target of AMPK and are reinforced by its activation.

## 6. Molecular Basis of the Effects of AMPK on AJCs: Implication of Novel AMPK Effectors in AJCs and AMPK-Driven Epithelial Transcription Factors

Since these early studies, there has been a general consensus that AMPK has a role in reinforcing TJ function and/or establishing apicobasal polarity in a variety of epithelial cells both in vitro and in vivo [76,77,78,79,80,81,82,83,84,85]. Although the molecular mechanisms underlying these phenomena are not yet completely elucidated, recent identification of novel AMPK effectors in AJCs and AMPK-driven epithelial transcription factors have improved our knowledge.

**Figure 5:**
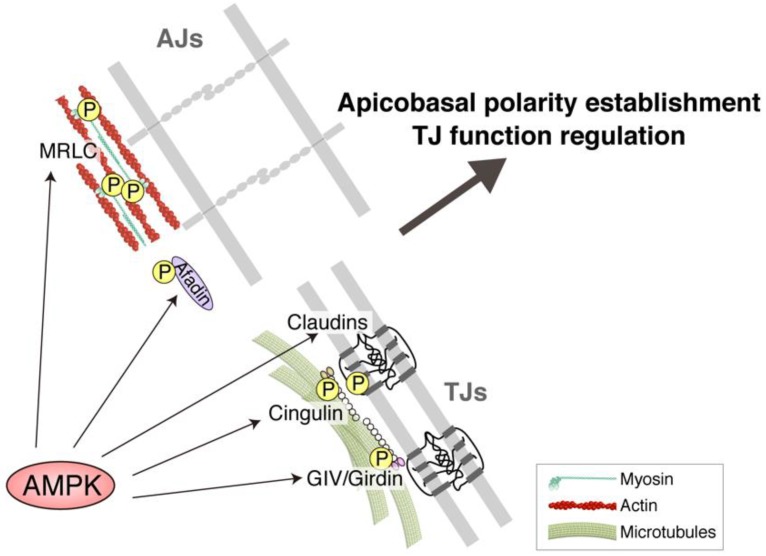
AMP-activated protein kinase (AMPK)-mediated regulation of apicobasal polarity establishment and tight junction (TJ) function are at least partially dependent on its phosphorylation of its substrates in AJCs. AMPK has been shown to phosphorylate myosin regulatory light chain (MRLC), cingulin, G-alpha interacting vesicle associated protein (GIV)/Girdin, claudins, and afadin, which can explain the role of AMPK in apicobasal polarity establishment and tight junction function regulation, at least partially. P, phosphate; AJs, adherens junctions.

To date, myosin regulatory light chain (MRLC), afadin, cingulin, claudins, and G-alpha interacting vesicle associated protein (GIV)/Girdin were identified as AMPK effectors in AJCs. The first one identified was MRLC, which was originally shown in 2007 to be directly phosphorylated at Thr-21 by AMPK using in vitro phosphorylation assays [36]. In that report, it was shown that this phosphorylation of MRLC is critical in the phenotype of AMPK-null mutants in *Drosophila* which displays severe abnormalities in apicobasal cell polarity [36]. However, it should be noted that this finding has since been questioned because this phosphorylation site does not match the optimal AMPK substrate motif found in all other established AMPK substrates [86]. In 2011, afadin was identified as the second AMPK substrate in AJCs by the study investigating adhesion-related molecules, which are important in AMPK-dependent TJ assembly [87]. Prior to that study, afadin, together with another cell adhesion molecule nectin, was known to constitute an important adhesion systems in epithelial cells [88], and nectin–afadin-based adhesion was also known to appear during the earliest stages in the process of epithelial morphogenesis prior to the expression of TJ proteins [89]. In that study, it was found that afadin is a direct substrate for AMPK using in vitro phosphorylation assays. It was also found that AMPK activation by AICAR treatment increases the interaction of afadin with ZO-1 and that knocking down afadin expression prevented AMPK-mediated facilitation of TJ assembly in MDCK II cells, suggesting the possibility that AMPK reinforces nectin–afadin-based adhesion at an early stage of cell–cell junction establishment to facilitate TJ assembly [87]. The third AMPK effector in AJCs was identified in 2013, during the search for microtubule-binding proteins in the AJC fraction, when we found that cingulin is a direct AMPK substrate and AMPK-mediated phosphorylation of cingulin facilitates cingulin-mediated linkages of apical microtubule networks to TJs in epithelial cells [80,90]. Cingulin is a specific TJ protein [91] and has two potential evolutionally conserved AMPK target motifs at residues Ser-132 and Ser-150, and we showed that wild-type cingulin can be directly phosphorylated by AMPK at both sites using in vitro phosphorylation assays. Furthermore, we showed that AMPK-mediated cingulin phosphorylation strengthens the interaction of cingulin with microtubules in mouse mammary gland epithelial Eph4 cells and is important in epithelial three-dimensional morphogenesis [80,92]. The fourth group of AMPK effectors in AJCs is the claudins. In 2014, it was shown that AMPK activation by AICAR increases phosphorylation of claudin-4 at Ser-199 in an extracellular signal-regulated kinase 1/2-dependent manner in SV40 immortalized rat submandibular acinar cell lines [93]. Shortly after this, it was shown that claudin-1 can also be directly phosphorylated at Thr-191 by AMPK using in vitro phosphorylation assays and that this phosphorylation may potentially be important in TJ reinforcement in epithelial Eph4 cells [94]. The last AMPK effector in AJCs discovered so far was GIV/Girdin, which was identified as a direct AMPK substrate in 2016 [95]. Prior to that work, GIV/Girdin was shown to be a guanine-nucleotide-exchange factor (GEF) for Gαi3 in trimeric G proteins and to play an important role in TJ formation and establishment of apicobasal epithelial cell polarity [96]. In that work, it was found that GIV/Girdin has an evolutionally conserved, potential AMPK phosphorylation motif at Ser-245 in the N-terminus of GIV/Girdin and that AMPK directly phosphorylates GIV/Girdin at Ser-245 using in vitro phosphorylation assays. It was also shown that, during apicobasal cell polarity establishment in MDCK II cells, GIV/Girdin becomes phosphorylated by AMPK and subsequently localizes at TJs through its binding affinity for the TJ-linked apical microtubule network. Further, it was shown that this phosphorylation of GIV/Girdin is essential in AMPK-dependent TJ formation and apicobasal epithelial cell polarity establishment, and that an oncogenic mutant of GIV which cannot be phosphorylated by AMPK increases tumor cell growth through the defects in establishing apicobasal cell polarity [95]. These results collectively show that there are several AMPK substrates in AJCs (MRLC, cingulin, claudins, and GIV/Girdin) and that the phosphorylation of these substrates impacts TJ function and apicobasal cell polarity, although how AMPK coordinates these different pathways merits future investigation (Figure 5).

**Figure 6:**
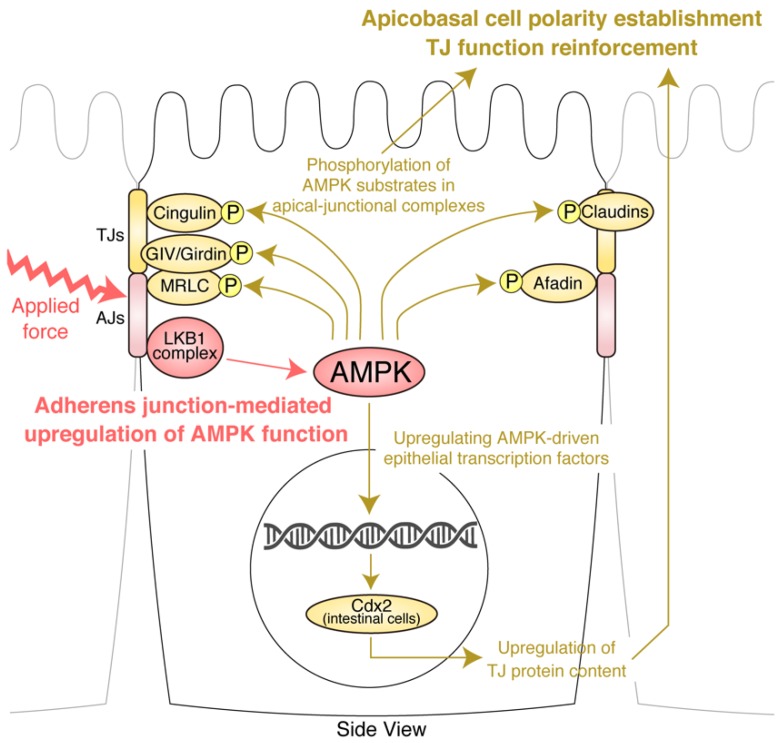
Overview of the reciprocal association between apical junctional complexes and AMP-activated kinase (AMPK). Adherens junctions (AJs) activate the liver kinase B1 (LKB1)–AMPK axis particularly in response to applied junctional tension. AMPK reinforces tight junction (TJ) function and promotes apicobasal cell polarization establishment through AMPK effectors in AJCs and AMPK-driven epithelial transcription factors.

Another important mechanism explaining the association between AMPK and TJs was described in 2017 [97]. Prior studies suggested the possibility that AMPK upregulates the expression of TJ proteins, thereby exerting a positive effect on TJ function; however, the underlying mechanism remained unknown [81,84]. In that study, it was found that, in intestinal epithelial Caco-2 cells, AMPK activation by AICAR upregulates expression of caudal type homeobox 2 (CDX2), a key transcription factor governing the differentiation of intestinal epithelial cells. It was also shown that this upregulation is caused by AMPK-dependent recruitment of methylase PRC2 and demethylase LSD1 to induce histone modifications of the CDX2 promoter. Furthermore, it was shown that intestine-conditional AMPK-knockout mice displayed impairment of intestinal epithelial differentiation and barrier function with a significant reduction in CDX2 expression [97]. Subsequent research found that AMPK activation by purple potato extract (PPE) in Caco-2 cells upregulates TJ protein expression in correlation with CDX2 expression and that AMPK-knockout abolishes the positive effects of PPE on CDX2 expression and TJ protein expression [98]. These findings suggest that, in addition to the AMPK substrates in AJCs, an AMPK-driven epithelial transcription factor plays an important role in AMPK-dependent TJ reinforcement via upregulation of TJ protein content (Figure 6).

## 7. Role of AMPK in Endothelial Barrier Function

Thus far, we have mainly focused on the role of AMPK in epithelial AJCs. Another intriguing aspect of AMPK is that it also has been shown to enhance endothelial TJ function [99,100,101,102]. An endothelial cell monolayer lines the inside of the intima of all blood and lymphatic vessels, forming a semipermeable barrier between the interstitial fluids within tissues and circulating fluids [103]. In general, TJs together with AJs form pericellular zipper-like structures between endothelial cells to restrict free diffusion of molecules, but the specific architecture and composition varies in different vascular beds. For example, TJs are more sophisticated in small arterioles, especially in highly specialized vascular beds like the blood–brain barrier (BBB) and inner blood–retinal barrier, whereas AJs are more prominent in postcapillary venules [103,104,105].

The overall molecular composition of endothelial TJs and AJs are similar to those of epithelial cells, with some small differences [103]. For example, the cadherin-family protein in epithelial AJs is E-cadherin, whereas it is vascular endothelial-cadherin (VE-cadherin) in endothelial AJs. It is noteworthy that N-cadherin is another major cadherin-family protein expressed in endothelial cells, but is excluded from endothelial AJs for an unknown reason [106,107]. N-cadherin has been shown to be important in the interactions of endothelial cells with pericytes and smooth muscle cells, which are thought to play an essential role in the maturation and stabilization of endothelial cells [108,109]. Interestingly, in 2011, using a pulmonary microvascular endothelial cell (PMVEC) wound healing model, it was found that AMPK-mediated TJ reinforcement is critical for PMVEC repair and that AMPK colocalizes with N-cadherin in PMVEC [99]. Following this finding, the biological importance of the AMPK–N-cadherin interaction was investigated using the same PMVEC wound healing model, and it was found that a short-hairpin RNA which decreases the expression of N-cadherin causes translocation of AMPK away from the membrane and inhibits AMPK-mediated barrier repair [110]. These findings collectively suggest that the N-cadherin–AMPK axis may be important for endothelial barrier assembly, but further studies are warranted for validation of these intriguing findings.

## 8. Therapeutic Potential of Targeting AMPK for Manipulation of Epithelial Barrier Function

One of the fascinating features of AMPK is that it is “druggable”. Indeed, we can artificially activate AMPK either directly (direct activator) or indirectly through AMP or calcium accumulation (indirect activator) using preexisting food compounds such as polyphenols and α-lipoic acid, as well as drugs such as metformin, thiazolidinediones, AICAR, and A79662 (summarized in Table 1). This leads to the question of whether these agents have any potential as therapeutics for diseases caused by barrier dysfunction. The answer may be “yes”.

By improving intestinal epithelial barrier dysfunction, metformin-induced activation of AMPK can alleviate inflammatory bowel disease in mouse models induced by interleukin-10 deficiency, dextran sulfate sodium, and heat stress [84,85,124]. Metformin-induced intestinal barrier reinforcement was also shown to alleviate fructose-induced non-alcoholic fatty liver disease by blocking translocation of bacterial endotoxin [78]. Moreover, it was shown that metformin-induced AMPK activation may inhibit respiratory infection via reinforcement of airway epithelial barriers [79], attenuate the severity of ischemia-induced renal tubular injury by facilitating TJ assembly in renal epithelial cells [77], and facilitate recovery from acute ischemic stroke by attenuating BBB disruption [102,125] (metformin is an indirect AMPK activator that has other AMPK-independent effects; however, these protective effects of metformin on epithelial barrier function was shown to be dependent on AMPK [77,78,79,84,85,102,124,125]). Furthermore, AICAR-induced AMPK activation was also shown to alleviate lipopolysaccharide (LPS)-induced lung injury via restoring pulmonary endothelial barrier integrity [110], and LPS-induced cardiac dysfunction by attenuating left ventricular wall edema [100]. Therefore, these results collectively show that AMPK is a promising therapeutic target for epithelial/endothelial barrier manipulation. However, it should be noted that AMPK has wide variety of function in wide variety of organs with its activity being spatiotemporally regulated. Therefore, systemic and uniform AMPK activation would cause too many off-target effects. AMPK subunits (α, β, or γ) occur as multiple isoforms encoded by distinct genes (α1 or α2; β1 or β2; γ1, γ2, or γ3), giving rise to 12 possible AMPK α/β/γ heterotrimers that are differently expressed in different tissues and exert different effects [47]. For example, the α1/β2/γ1 heterotrimer is predominant in intestinal epithelial cells [126] and, while α1/β2/γ1, α2/β2/γ1, and α1/β2/γ3 heterotrimers are present in skeletal muscles [127,128], only the α2/β2/γ3 heterotrimer is activated during high intensity exercise [128,129]. Most AMPK activators/inhibitors that are commonly used today are not isoform-specific; therefore, further elucidation of isoform specific localization and/or function, as well as further development of isoform-specific activators and inhibitors, is important for AMPK-based organ- and/or barrier-specific therapy.

## 9. Conclusions

AMPK is a well-known central regulator of energy metabolism. As we have reviewed here, it has also been shown over 15 years that AMPK has a reciprocal association with AJCs, and fine-tunes TJ function and apicobasal cell polarity establishment in epithelial/endothelial cells. The complete molecular mechanisms underlying these phenomena have not yet been elucidated; however, identifications of novel AMPK effectors in AJCs and AMPK-driven epithelial transcription factors have enhanced our knowledge, and recent technological advances will contribute to better understanding. For example, recent development of transgenic mice expressing a highly sensitive fluorescence resonance energy transfer-based biosensor would visualize dynamic AMPK responses in vivo [130,131]. The formation and regulation of AJC-mediated epithelial cell–cell adhesion and paracellular barrier are critical for the biological systems with its disruption being implicated in various diseases; therefore, further elucidation of molecular details underlying an association between AMPK and AJCs will contribute to the better understanding of epithelial cell physiology as well as the development of therapies, enabling us to manipulate epithelial/endothelial barrier function.

## Figures and Tables

**Table 1:** List of commonly-used AMPK activators and inhibitors.

	Compound Name	Mechanism	Isoform Selectivity	Reference
Indirect activators	Metformin (biguanides)	Inhibiting the mitochondrial respiratory chain Complex I → Increase in AMP/ATP ratio	No	[111,112]
	Troglitazone (thiazolidinediones)	Inhibiting the mitochondrial respiratory chain Complex I → Increase in AMP/ATP ratio	No	[112,113]
	Resveratrol (polyphenols)	Inhibiting F1F0–ATPase/ATP synthase → Increase in AMP/ATP ratio	No	[112,114]
	Quercetin (polyphenols)	Inhibiting F1F0–ATPase/ATP synthase → Increase in AMP/ATP ratio	No	[112,115]
	α-Lipoic acid	Increasing calcium level?	No	[116]
Direct activators	5-Aminoimidazole-4-carboxamide ribonucleoside (AICAR)	Prodrug, converted to 5-amino-1-β-d-ribofuranosylimidazole- 4-carboxamide-5′-monophosphate (ZMP) → ZMP binds to site-3 in the γ-subunit	No	[112,117]
	Compound-13 (C-13)	Prodrug, converted to Compound-2 (C-2) → C-2 binds to the γ-subunit	α1 > α2	[118]
	A-769662	Binding to the allosteric drug and metabolite (ADaM) site → Allosteric activation	β1 > β2	[56,119]
	Compound 991	Binding to the ADaM site → Allosteric activation	β1 > β2	[56]
	Salicylate	Binding to the ADaM site → Allosteric activation	β1	[120]
	MT63-78	Binding to the ADaM site → Allosteric activation	β1	[121]
Inhibitors	Compound C (dorsomorphin)	Binding to the α-subunit → Competitive inhibition of ATP	No	[111,122]
	MT47-100	Binding to the β2-subunit → Allosteric inhibition	β2 (β1 activation)	[123]
	SBI-0206965	Binding to the α-subunit → Mixed-type inhibition of ATP	No	[65]

## Abbreviations

TJs	Tight junctions
AJs	Adherens junctions
AMP	Adenosine monophosphate
AMPK	AMP-activated protein kinase
HMG-CoA	3-hydroxy-3-methylglutaryl-coenzyme A
acetyl-CoA	Acetyl-coenzyme A
ATP	Adenosine triphosphate
LKB1	Liver kinase B1
CaMKKβ	Ca^2+^/calmodulin-dependent protein kinase kinase β
PJS	Peutz–Jeghers syndrome
MDCK	Madin–Darby canine kidney
FLCN	Folliculin
BHD	Birt–Hogg–Dubé
AICAR	5-Aminoimidazole-4-carboxamide ribonucleoside
MRLC	Myosin regulatory light chain
GIV	G-alpha interacting vesicle associated protein
GEF	Guanine-nucleotide-exchange factor
CDX2	Caudal type homeobox 2
PPE	Purple potato extract
BBB	Blood–brain barrier
VE-cadherin	vascular endothelial-cadherin
PMVEC	Pulmonary microvascular endothelial cell
ZMP	5-amino-1-β-d-ribofuranosylimidazole- 4-carboxamide-5′-monophosphate
LPS	Lipopolysaccharide
